# Generation of a Free-Living Ground-Truth Validation Dataset for Wearable Measures of Physical Activity, Sedentary Behavior, Sleep, and Heart Rate in Adults (OxWEARS): Protocol for a Cross-Sectional Study

**DOI:** 10.2196/78779

**Published:** 2025-12-29

**Authors:** Benjamin D Maylor, Scott R Small, Tatiana Plekhanova, Laura Brocklebank, Stefan van Duijvenboden, Rachel Sharman, Elizabeth A Hill, Fredrik Karpe, Simon D Kyle, Aiden Doherty

**Affiliations:** 1 Nuffield Department of Population Health, University of Oxford, Old Road Campus, Oxford, OX3 7LF, United Kingdom, 44 1865 617794; 2 Li Ka Shing Centre for Health Information and Discovery, Big Data Institute, University of Oxford, Oxford, United Kingdom; 3 Nuffield Department of Clinical Neurosciences, Sir Jules Thorn Sleep and Circadian Neuroscience Institute (SCNi), University of Oxford, Oxford, United Kingdom; 4 School of Applied Sciences, University of the West of England, Bristol, United Kingdom; 5 Oxford Centre for Diabetes, Endocrinology and Metabolism, University of Oxford, Oxford, United Kingdom; 6 NIHR Oxford Biomedical Research Centre, Oxford University Hospitals NHS Trust, Oxford, United Kingdom

**Keywords:** validation, wearables, machine learning, physical activity, sedentary behavior, sleep, heart rate

## Abstract

**Background:**

Wearable devices enable continuous measurement of physical activity, sedentary behavior, sleep, and heart rate under free-living conditions. However, most validation studies rely on small, homogeneous samples; are conducted under laboratory conditions; or lack gold standard ground-truth measurements, limiting the generalizability and accuracy of derived metrics. There is a pressing need for open-access, large-scale, free-living validation datasets that include multisensor data from diverse body locations and participant demographics to aid in model development.

**Objective:**

The Oxford Wearable ECG, Activity, Circadian Rhythm, and Sleep Validation Study (OxWEARS) aims to (1) validate accelerometer-based measurement of physical behaviors across 5 body sites against annotated camera data; (2) validate measurements of sleep and sleep staging from 5 different body sites against polysomnography; (3) validate wrist-worn photoplethysmography heart rate measurements against chest-worn electrocardiogram; and (4) generate a comprehensive, annotated, and anonymized dataset for open-access research use.

**Methods:**

This cross-sectional study will recruit approximately 160 adults (aged ≥40 years) stratified by age, sex, and BMI from the Oxford BioBank. Over 3 days and 4 nights, participants will wear sensors on the wrists, chest, hip, thigh, and ankle. Ground-truth measures will be obtained from a chest electrocardiogram patch for heart rate, a first-person camera for activity annotation, an ankle-worn accelerometer for step count, and at-home polysomnography for sleep. An under-mattress sensor will collect measures of sleep, respiration rate, and bedtime, and a subjective sleep diary will also be obtained. Signals from different wear locations will be compared against the ground truth using precision, recall, *F*_1_-score, κ, and agreement metrics.

**Results:**

Recruitment commenced in November 2024, with 15 participants enrolled by May 2025. Overall, 50% of eligible adults contacted were happy to consent to the study, with excellent compliance with the protocol observed to date. Data collection is ongoing and expected to conclude in 2026, with the final annotated dataset made publicly available as soon as possible thereafter.

**Conclusions:**

The OxWEARS study will generate an openly accessible dataset containing more than 10,000 annotated hours from a stratified sample of adults. This will directly support scalable, generalizable human activity recognition efforts, while also enabling robust development and benchmarking of wearable-derived health metrics.

## Introduction

The use of wearable devices for the assessment of physical activity, sedentary behavior, and sleep allows for the quantification of 24-hour human physical behaviors without the recall and social desirability biases associated with self-reported measures [1-3]. While these behaviors are commonly measured using commercial- and research-grade devices within epidemiological studies, the accuracy of the derived metrics against gold standard ground truth is currently uncertain. To date, most activity and sleep validation studies of wearables have been of poor quality—conducted in small, homogeneous cohorts; under laboratory conditions for a single day; and often without a gold standard reference [4,5]. Additionally, most studies focus on either sleep or physical activity, resulting in a lack of ground-truth reference measures for all behaviors across the 24-hour day.

While some accelerometer models have been trained using free-living validation data, significant room for improvement exists in model generalizability and specifically in improved performance in analyzing physical activity, sedentary behavior, and sleep. For example, some validation studies have collected wrist-based accelerometer data paired with first-person camera images for behavior classification [6,7], and these methods have subsequently been adopted in large epidemiological studies for health research [8,9]. However, these data were collected in a convenience sample over a single day without deference to age, sex, and BMI [10]. Additionally, more multisensor datasets are needed to compare data collected concurrently from different wear locations, as reflected in the existing literature. For example, large datasets exist with accelerometers worn on the wrist [8,11], hip [12], and thigh [13-15]; however, there is limited evidence regarding the harmonization of physical behavior phenotypes generated from different wear locations for subsequent statistical analyses. Furthermore, combining different signals from newer devices, such as photoplethysmography (PPG) and accelerometry, may improve the detection of sleep, physical activity, and sedentary behavior by providing additional data streams for model development. Finally, in most validation studies, raw data are not published, hampering external validation efforts and method development to improve performance [4,16].

Therefore, our overarching aim is to collect a free-living validation dataset, annotating wearable sensor data with gold standard, ground-truth labeled data, for application to large-scale accelerometer datasets with linked health records.

The main aims of this study are as follows:

1. To validate the measurement of physical activity and sedentary behavior from accelerometers worn on the wrist, chest, waist, hip, thigh, and ankle compared with ground-truth annotations from wearable camera data.
2. To validate the measurement of sleep and sleep staging from accelerometers worn on the wrist, chest, waist, hip, and thigh compared with gold standard polysomnography (PSG) sleep metrics.
3. To validate the measurement of heart rate and heart rate variability from wrist-worn PPG sensors against a ground-truth chest-worn electrocardiogram (ECG) patch.
4. To generate an anonymized dataset of annotated physical activity, sedentary behavior, sleep, and cardiac monitoring data for unrestricted use by the wider scientific community.

## Methods

### Ethical Considerations

Ethics approval was received from the University of Oxford Central University Research Ethics Committee on August 1, 2023 (R74559). Before the initiation of any study procedures, all participants were provided with an information sheet (Multimedia Appendix 1) and gave written informed consent using a digital signature platform compliant with ISO 27001 standards (E-Sign; E-Sign Ltd). Participants’ privacy is of the utmost importance to us; therefore, no identifying information will be published, and individual data will be deidentified before any data release. Participants will receive no compensation for their participation but will be provided with a nondiagnostic report on their sleep and physical behaviors during the course of the monitoring period. Conforming to current ethical frameworks [17,18], written consent to be recorded by the video camera is not required from bystanders; however, participants will be instructed to obtain verbal permission from and provide an explanation of the device to family members, cohabitants, workplace managers or supervisors, or other people in settings where a reasonable expectation of privacy exists. This approach was considered reasonable. A script and information card will be provided for use if participants are questioned by members of the public. While we obtain consent, we will also ask participants to sign a privacy agreement for the recording of video for this study in public and private settings (Multimedia Appendix 2). Participants will be instructed on how to cover the camera or pause data collection at any time when they need privacy or feel uncomfortable or unsafe. Examples of this include using the bathroom (in public or private), using public changing rooms, interacting with unrelated children, or working in contexts where intellectual property must be protected. In line with the ethical framework established by Kelly et al [17], participants can request the removal of any unwanted or sensitive footage potentially caught on camera. Privacy concerns related to the wearable camera data are of critical importance. Therefore, raw video footage will never be released, and any examples used (such as for annotator training) will be confined to the University of Oxford research team. All data will remain on University of Oxford servers.

### Data Deidentification

In line with previous research, no wearable camera data will be shared outside of the University of Oxford [7]. Each participant will be assigned a unique study identifier, which will be used to label all associated raw data files. Because participants could potentially be deidentified based on combined age, sex, and BMI, we will report only the subgroup to which each participant belongs, as described earlier. Finally, the start dates of the raw data will be randomized, and time stamps will also be shifted by a small random amount so that they do not reflect the true dates and times of the data. Similar steps have been used previously to ensure deidentification of participants taking part in studies [7].

### Recruitment

OxWEARS is a cross-sectional study and will recruit a target sample of 160 participants from the Oxford BioBank [19]. A sample size of 160 will provide 80% power to detect a difference of 0.05 in Cohen κ score (effect size=0.25, assuming an SD of 0.25 from previous studies [20]) between 3 age groups using a fixed effects one-way ANOVA test (α=.05). The Oxford BioBank is a population-based cohort of approximately 9000 participants aged 25 to 55 years recruited in Oxfordshire, United Kingdom, from 1999 onward. Inclusion and exclusion criteria for this study are presented in Textbox 1. Recruitment will target the creation of a final study cohort evenly balanced by sex, age (40‐54 years, 55‐64 years, and ≥65 years), and BMI (18.5‐25 kg/m^2^, 25‐30 kg/m^2^, and >30 kg/m^2^). The distribution of invitations will be regularly rebalanced to achieve 13 to 14 participants in each subgroup by the end of the study.

Textbox 1. **Inclusion and exclusion criteria.**

**Inclusion criteria**

- Enrolled participant of the Oxford BioBank
- Healthy adults aged 40 years and older
- Able to ambulate (with or without a walking aid)
- Willing to wear multiple sensors over 3 days and 4 nights of data collection

**Exclusion criteria**

- Unable to speak or understand English
- Self-reported neurological conditions or diagnosed sleep impairment
- Self-reported clinicians, teachers, caregivers, or anyone working in environments where image capture would be inappropriate and who cannot commit to 3 consecutive days of image capture outside of these sensitive environments
- Unwilling to wear all the monitors according to the study protocol

### Study Timeline

**Figure 1.**
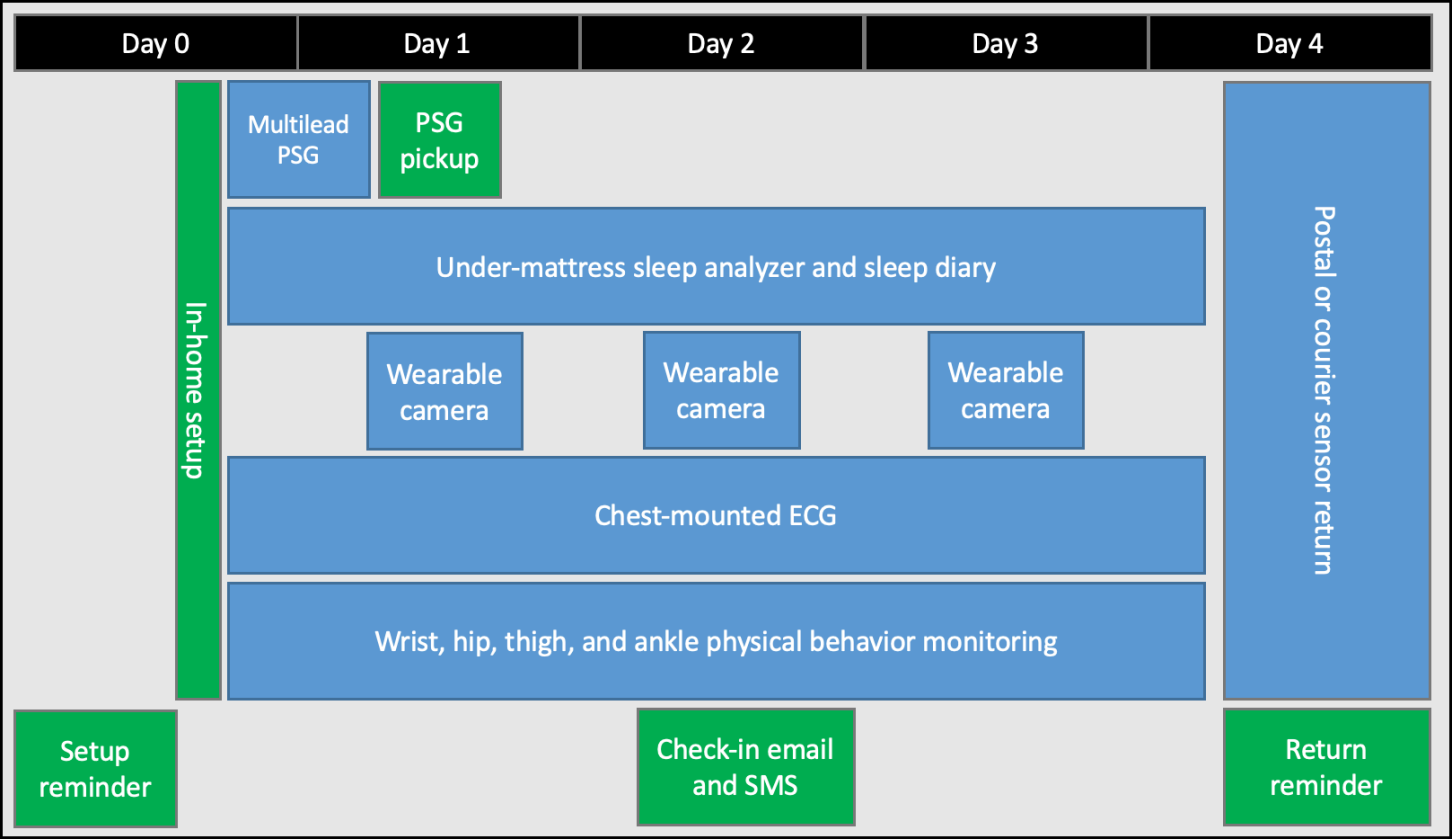
Overview flowchart of study activities during the data collection window. Green activities are conducted by the OxWEARS research team, and blue activities are conducted or collected by the study participant. ECG: electrocardiography; PSG: polysomnography.

**Figure 2.**
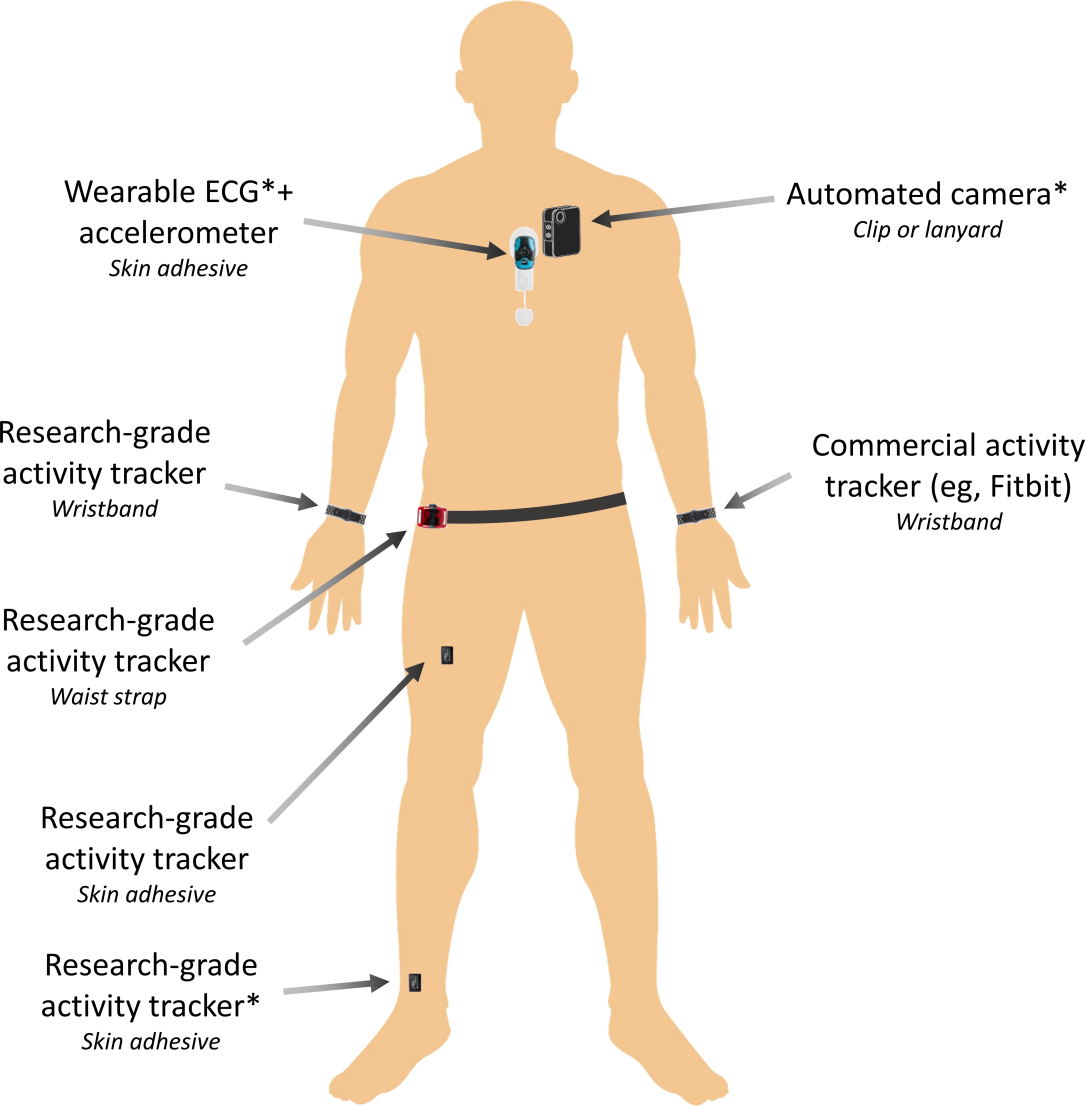
Distribution of wearable sensors for 3-day and 4-night ambulatory monitoring, including wrist, waist, thigh, and ankle accelerometers; a chest-mounted ECG and accelerometer patch; and an automated camera. *Denotes ground-truth or reference measurements (image designed by Freepik).

A study timeline is presented in Figure 1, outlining the overall data collection period. Data collection will be conducted in the free-living environment for 3 full days and 4 consecutive nights. Following receipt of consent, an in-person study setup visit is arranged, wherein 2 members of the research team will meet the participant at their home to set up and deploy the wearable sensors, camera, and under-mattress sleep analyzer for the data monitoring period, in addition to the PSG sleep assessment for the first night only. One night of PSG was chosen to balance participant and researcher burden with the inclusion of a gold standard sleep assessment. The distribution of ambulatory wearable sensors across the body is presented in Figure 2, with further description in the following methodological subsections.

### Ground-Truth ECG Data Collection

Ground-truth cardiac monitoring will be conducted using a clinical-grade single-lead wearable ECG patch (Bittium Faros 180; Bittium) affixed in a vertical orientation directly to the skin on the chest immediately inferior to the jugular notch and superficial to the sternum. Before adhesion, participants with chest hair will be instructed to shave the designated area to ensure adequate adhesion as per manufacturer guidelines. Continuous single-lead ECG data will be collected at 250 Hz and logged directly onto the ECG device for the entirety of the monitoring period (24 hours/day). In addition to raw ECG data, the monitor will be set to record continuous raw triaxial accelerometer data at the chest at a sampling rate of 100 Hz and with a dynamic range of ±4*g*.

### Ground-Truth Physical Behavior Data Collection

To obtain ground-truth data on everyday activities, participants will wear a first-person perspective video camera (MIUFLY) during waking hours for 3 days, mirroring procedures from prior studies using first-person image capture to annotate physical activity and sedentary behavior [7,21]. In this study, a wearable camera is worn by the participant via a chest harness, neck mount, clip, or lapel and continuously records video during participant waking hours. Previous automated camera studies of this nature have reported wear time compliance of more than 80% [22,23]. No audio is captured by the camera, and all footage is encrypted on the device so that only the research team can download and view the video. Participants will be instructed on how to cover the camera or pause data collection at any time when they desire privacy or feel uncomfortable or unsafe. Examples of this include using the bathroom (public or private), public changing rooms, interacting with unrelated children, or working in contexts where intellectual property must be protected.

### Ground-Truth Sleep Assessment

On the first study night, ground-truth sleep data will be collected with an ambulatory diagnostic multichannel PSG system (SOMNO HD Eco; S-Med Limited). The electroencephalogram (EEG) array will be attached in accordance with the 10-20 system of electrode placement [24]. The montage and recording settings (30-second epochs, 256 Hz sampling rate for EEG channels) will conform to the American Academy of Sleep Medicine (AASM) recommendations [25]. The following channels will be recorded: scalp EEG (F3, F4, C3, C4, O1, and O2), bilateral references on the mastoid processes (M1 and M2), grounding electrode (placed on FpZ), a common scalp reference electrode (Cz), bilateral electro-oculogram, 2-lead ECG, and 3-lead submental electromyography. Participants will be asked to change into sleeping clothes prior to sensor setup to minimize the risk of electrode disturbance. Prior to recording, researchers will check signal quality via a tablet and replace electrodes with high impedance values (>5 kΩ). The in-home sleep study will be conducted for 1 night only. Participants will be instructed to blink several times to indicate lights off at the time of their choosing. Participants will be instructed on how to remove the electrodes themselves following sleep, and a member of the research team will make a return visit the morning following the at-home visit to collect the PSG equipment and answer any further questions regarding the study.

### Wearable-Based Physical Behavior Monitoring

Participants will be asked to wear 5 stand-alone wearable devices continuously (24 hours/day) throughout the course of the study period (Figure 2). The wear locations for the sensors were selected based on their popularity in previous validation studies [4,5]. The recording specifications of each device and sensor are detailed in Table 1. Wrist-measured physical behavior data will be collected using a combination of research-grade and consumer-grade devices. Specifically, a multimodal research-grade device (ActiGraph LEAP: ActiGraph LLC) consisting of triaxial accelerometer, gyroscope, and PPG sensors will be worn on the participant’s dominant wrist. Similarly, a consumer-grade device (Fitbit Sense 2; Fitbit by Google) with comparable multimodal sensors to the ActiGraph LEAP will be worn on the participant’s nondominant wrist, according to manufacturer’s instructions.

**Table 1.** Wearable device data capture.

Device	Location	Sensors and recording settings	Heart rate
Bittium Faros 180	Chest	Accelerometer: 100 Hz, ±4*g*	1-channel ECG^a^: 250 Hz
ActiGraph LEAP	Dominant wrist	Accelerometer: 32 Hz, ±8*g*; gyroscope: 128 Hz and ±2000 dps^b^	PPG^c^: green (530 nm), 128 Hz
Fitbit Sense 2	Nondominant wrist	60 s epoch proprietary phenotypes	60 s epoch heart rate average
ActiGraph GT3X-BT	Waist or hip	Accelerometer: 100 Hz, ±8*g*	^d^—
Axivity AX6	Dominant thigh	Accelerometer: 100 Hz, ±8*g*; gyroscope: 100 Hz, ±2000 dps	—
Axivity AX6	Dominant ankle	Accelerometer: 100 Hz, ±8*g*; gyroscope: 100 Hz, ±2000 dps	—

a ECG: electrocardiogram.

b dps: degree per second.

c PPG: photoplethysmography.

d Not applicable.

Participants will wear an ActiGraph GT3X-BT monitor (ActiGraph LLC) on their hip continuously throughout the data collection period, removing it only for water-based activities. The device will be worn using an adjustable belt strap, with a buckle for easier removal.

For thigh monitoring, a medical-grade hypoallergenic adhesive dressing will be used to affix an accelerometer (Axivity AX6; Axivity Ltd) on the anterior aspect of the participant’s dominant thigh, wrapped in a nitrile sleeve for protection. The Axivity AX6 houses a triaxial accelerometer and triaxial gyroscope, which will both record at a sampling frequency of 100 Hz and dynamic sensor ranges of ±8*g* and ±2000 degrees per second, respectively.

As an additional measurement point for physical activity, a further Axivity AX6 device (with matching recording characteristics) will be mounted on the dominant-side ankle, wrapped in a nitrile sleeve using medical adhesive tape. This is of specific interest for potential step count validation [26], for which a separate study including video-captured daily steps with concurrent ankle- and wrist-worn Axivity AX6 devices will be conducted to assess the validity of the ankle as a suitable ground-truth measure for step count in 50 adults, incorporating machine learning methods to estimate step count compared with proprietary algorithms assessed previously [26]. This model will then be applied to OxWEARS to compare step count from the ankle with each of the other wear locations (chest, both wrists, hip, and thigh).

### Nearable-Based Sleep Assessment (Under-Mattress Sensor)

In addition to the ground-truth PSG sleep assessment, a popular consumer-grade sleep device (Withings Sleep Analyzer; Withings) will be set up by the researcher for the full 4 nights of the study. The Withings Sleep Analyzer will be placed under the participant’s mattress in line with where the heart would be while the participant lies in bed. It uses ballistocardiography and a built-in microphone to estimate sleep duration, efficiency, sleep onset latency, sleep staging (awake, light, deep, or rapid eye movement sleep), as well as breathing rate and snoring events, using proprietary algorithms. It has shown some early promise as a potential “nearable” device for individuals to monitor long-term sleeping patterns without the need to physically wear a device [27].

### Subjective Sleep Assessment

As a final complementary measure of sleep duration and quality, participants will additionally be asked to complete a standardized self-report sleep diary on each of the 4 study nights [28], which captures times the participant got into bed, went to sleep, woke up, and got out of bed. We have further adapted this to capture information on perceived sleep quality and whether any naps were taken outside of the primary sleep window (Multimedia Appendix 3).

### Data Processing and Analysis

Physiological reference metrics derived from the ground-truth clinical-grade cardiac monitor will include participant heart rate and heart rate variability. There is ongoing work to develop and validate an ECG algorithm, which will be made publicly available in the future. During this process, clinical experts with extensive experience will review ECGs for RR intervals to determine algorithm performance. Derived heart rate and variability will be calculated on a per-epoch basis in 10-second windows and will serve as the ground-truth labels of heart rate for the study. Comparisons will then be made between these cardiac monitor–derived metrics and PPG-derived metrics [29] from both wrist-worn devices in terms of epoch-level accuracy and agreement across participant age, sex, and BMI subgroups.

Physical activity behaviors derived from the camera will take the form of video annotation of labeled activities based on posture (eg, lying, sitting, and standing) and whole-body movements (eg, walking, running, and cycling), in line with previous research [30]. Each participant’s camera data will be annotated by an annotator who has completed a rigorous training regimen and is certified for annotation after consistently reaching a κ agreement of >0.8 against a series of day-long reference participants. If multiple annotators are required, we will additionally assess for interrater reliability scores. Further annotation and assessment will be conducted against machine learning–based automated image and video annotations [31]. These annotations will serve as the basis for retraining machine learning behavior classification models to better identify periods of sedentary, light, moderate, and vigorous physical activity [9,32].

Ground-truth sleep metrics, including time of sleep onset, waking time, and sleep staging, will be scored according to the AASM 2023 guidelines [25] by a single AASM-accredited sleep scientist with additional European Sleep Research Society accreditation and more than 15 years of experience working with EEG data. Sleep stages (non–rapid eye movement sleep [stages N1, N2, and N3], rapid eye movement, and awake) will be assigned for every epoch (30 seconds). A subsample of recordings (10%‐20%) will be scored by a secondary accredited researcher to assess interrater reliability. As per recommendations in the field, scoring will be adjusted until interrater agreement reaches >80%. Similar to the physical activity behavioral ground-truth annotations, sleep annotations derived from the in-home PSG will serve as the basis for comparison of sleep time, sleep efficiency, and sleep staging derived from other accelerometers and the under-mattress sleep nearable, in addition to current accelerometer-based machine learning sleep models [33,34].

Data quality will be assessed regularly throughout the data collection period to check protocol adherence. This will involve processing the raw accelerometer data from the research-grade wearables through existing analytic software, such as Biobank AccelerometerAnalysis (University of Oxford) [8] and Stepcount (University of Oxford) [35], to assess device recording duration and nonwear detection based on accelerometer thresholds. The Fitbit- and Withings-derived data will be checked at the summary level to flag possible poor compliance. PSG data quality will be assessed within the manufacturers' software using an automated sleep-stage classification system. We will report on the technical validation of the data when we publish the complete dataset, including the number of participants and the volume of camera-labeled data, both separately and when combined with concurrent data from other devices.

The primary aim of this study is to collect a high-quality validation dataset to facilitate new ways of analyzing time-series wearable data. As such, there will be many future imaginative analyses of these data that we do not anticipate at present. At present, we anticipate that mean precision, recall, *F*_1_-score, Cohen κ, and accuracy will be used to evaluate model performance for all comparisons with ground-truth labels. Additionally, summary metrics will be assessed against the ground-truth measure using mean absolute bias, mean amplitude percentage error, Cohen κ [36], and Bland-Altman plots [37].

### Metadata Description

Our complete dataset and metadata will be hosted by the Oxford University Research Archive under the Creative Commons “Attribution 4.0 International (CC BY 4.0)” license. Raw accelerometry, ECG, and PPG data will be provided as compressed CSV files in folders for each participant. We will provide separate CSV files containing a dictionary of annotation labels for scored PSG data, a full annotation schema, labels for wearable camera data, and participant characteristics (age category, sex, and BMI category).

## Results

To date, 150 participant information sheets have been sent to members of the Oxford BioBank database. Of these, 30 (20%) have expressed interest, and 15 (50% of those expressing interest) have provided consent. Common reasons for not progressing to consenting so far include ineligibility due to occupation (teacher or health care worker), requests to be contacted at a later date, and inability to make contact with the participant. The first participant consented and completed data collection in November 2024. As of May 2025, we had enrolled 15 participants, with 12 (80%) completing the monitoring period with excellent adherence to the protocol. Data collection is expected to be completed in 2026.

## Discussion

This protocol details the design of a new free-living validation dataset to more accurately characterize physical behaviors and heart rate using wearable-based sensors on the wrist, chest, waist, hip, thigh, and ankle. By establishing ground-truth metrics for physical activity, sedentary behavior, sleep, and heart rate in a cohort of middle-aged and older adults, we attempt to create the largest resource for the validation of current and future methods for deriving physical behaviors and heart rate that is freely accessible to the wider research community. Furthermore, the methods that we implement in this study overlap with past research [7,38]; ongoing studies at the University of Oxford (S van Duijvenboden, unpublished data, December 2025); and future planned studies in low- and middle-income countries, such as India, South Africa, and Malaysia. This will foster efforts to improve the generalizability of machine learning models by training on diverse datasets, in addition to providing suitable datasets for external validation and pooled analyses. Large cohort studies currently hold rich datasets of unlabeled data. The creation of this curated dataset, comprising labeled behaviors for all popular sensor wear sites [39], will support the development of more accurate and novel phenotyping. The application of these phenotypes into epidemiological research will then be used to provide novel insights into human health, with respect to risk prediction, discovery of target mechanisms, and new methods to prioritize and assess the impact of potential treatments on day-to-day physical activity, sedentary behavior, and sleep.

Chest-worn ECGs are increasingly being used in cohort and clinical trials, where long-term monitoring of ECG data during free living can provide a more comprehensive understanding of heart function during daily activities, exercise, and sleep. The validation of ECG data from chest-worn wearables supports ongoing and future long-term cardiac monitoring trials such as the UK BioBank cardiac monitoring substudy [40]. At present, there is promising, but very limited, evidence on the validity of accelerometer-derived physical behavior phenotypes from chest-worn accelerometers based on simulated free living in small samples [41,42]. Incorporating physiological parameters, such as heart rate and other ECG parameters—key markers of cardiovascular health—could provide deeper insights into the intensity and impact of physical activity. For example, several activities performed at moderate-to-vigorous intensity, such as cycling, resistance exercises, or walking on an incline, are assessed with limited certainty with accelerometry [43] and are likely to be better captured by including heart rate monitoring, as it is not affected by the modality biases inherent to single-site accelerometry. Kuo et al [44] demonstrated an increase in reliability of 10% to 15% when including heart rate on top of accelerometry at 5 different wear sites to estimate energy expenditure during treadmill exercise at different speeds and gradients. This study was limited to a small sample of 16 young, healthy men and a treadmill protocol. Free-living behaviors captured in a larger, stratified sample, such as in this study, will further enhance our understanding of the added value of combining accelerometer data with heart rate, as these measures become increasingly available in research and consumer markets.

Identification of sleep stages that involve minimal movement may also benefit from additional physiological metrics to enhance their accuracy and reliability. This has been demonstrated previously in 31 adults who wore an Apple watch during a PSG assessment [45]. The combination of accelerometer with additional PPG sensor did not improve overall wake-sleep classification. However, sleep staging classification accuracy improved by 15% to 25% when heart rate was added as a feature on top of accelerometry signal alone and showed similar performance when applied to a large heterogeneous sample of approximately 6800 US adults from the National Sleep Research Resource [46]. This study will generate the largest accessible free-living dataset with ground-truth annotations for model development to improve human activity recognition at the chest for immediate application to clinical trial and prospective cohort datasets.

Strengths of this study include the large, stratified sample to create a more heterogeneous, multimodal dataset; the inclusion of ground-truth measures of physical activity, sedentary behavior, sleep, and heart rate; and the planned release of the data to the wider research community. One limitation of this study is the lack of dietary or cognitive data, meaning certain behaviors cannot be linked to physiological metrics, for example, postmeal sedentariness and heart rate variability. Future work could explore the capture of these behaviors to provide additional context to physiological responses. Furthermore, despite recruiting a large sample size in comparison to existing device validation studies, subgroup analyses may be challenging when assessing algorithm performance. However, due to our methodology, it would be possible for these data to be combined with other datasets (eg, Capture-24) to increase the statistical power of these comparisons in the future. Finally, although we recruit a sample stratified by age, sex, and BMI, our sample does not include younger adults aged <40 years, those with chronic illnesses, or non-English speakers. Therefore, the results will only be applicable to healthy adults with a demographic profile similar to the recruited participants. Future work should strive to include representativeness of these characteristics in addition to improving representativeness from low- and middle-income countries to assess generalizability of machine learning algorithms trained on this dataset. Despite these limitations, we anticipate that the study’s strengths will make the resulting dataset the largest and most comprehensive open-access validation dataset worldwide.

## Supplementary material

10.2196/78779 Multimedia Appendix 1 Participant information sheet.

10.2196/78779 Multimedia Appendix 2 Equipment and privacy agreement.

10.2196/78779 Multimedia Appendix 3 Sleep diary.

## Abbreviations

AASM: American Academy of Sleep Medicine
ECG: electrocardiogram
EEG: electroencephalogram
PSG: polysomnography
