# Supplementary material for: Generation of a Free-Living Ground-Truth Validation Dataset for Wearable Measures of Physical Activity, Sedentary Behavior, Sleep, and Heart Rate in Adults (OxWEARS): Protocol for a Cross-Sectional Study
Source: JMIR Res Protoc. 2025 Dec 29;14:e78779. doi: 10.2196/78779 (PMC12747664; doi:10.2196/78779)
Supplement: Multimedia Appendix 1 [file resprot-v14-e78779-s001.docx]

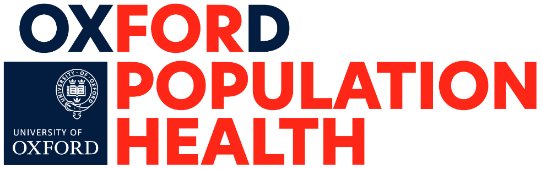

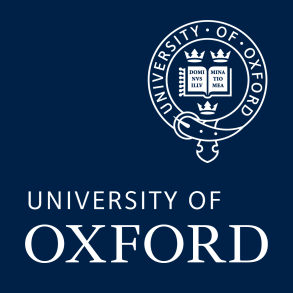


Professor Aiden Doherty
Nuffield Department of Population Health
Big Data Institute
aiden.doherty@ndph.ox.ac.uk

**PARTICIPANT INFORMATION SHEET**

OxWEARS: Oxford Wearable ECG, Activity, circadian Rhythm, and Sleep validation study

Wearable sensors, such as Fitbit-like devices, offer the opportunity to transform our understanding of the causes and consequences of disease. However, we don’t know how accurate wearables really are. We would therefore like to invite you to take part in a world-leading research study to figure out exactly how accurately wearables capture data in real-life. Data from this study could help us more accurately measure how well you sleep and how active you are.


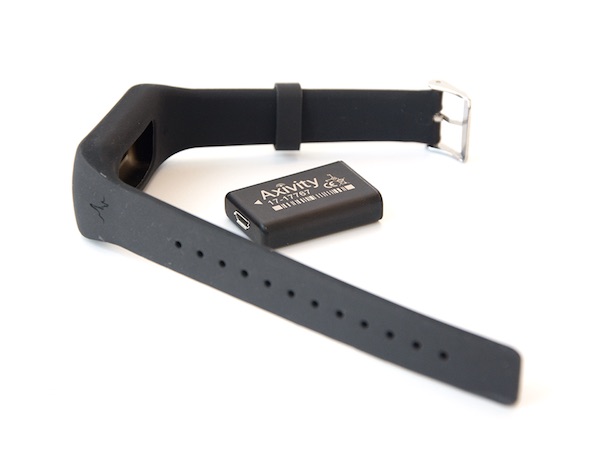


Before you decide, it is important that you understand why the research is being done and what it would involve for you. Please take time to read this information, and discuss it with others if you wish. *If there is anything that is not clear, or if you would like more information, please ask us.*

# What is the purpose of the study?

Wearable sensors are devices that can be worn on the body to track different aspects of a person’s health and daily activities. These sensors can help researchers and medical professionals understand how a person’s lifestyle and sleep patterns are related to their overall health: information that is particularly difficult to study in traditional hospital or laboratory settings. The goal of this study is to improve the techniques that researchers use to analyse data from wearable sensors. Data collected from participants in this study will be used to improve research methods using wearables sensors to assess physical activity, heart rate, and sleep. Improved methods and analysis techniques from this study will allow future researchers to investigate the link between lifestyle, sleep quality, and health outcomes like cardiovascular disease and Alzheimer’s disease.

# Why have I been invited?

- You have been invited because you are an active member of the Oxford Biobank research cohort
- We are looking for 200 participants from the Oxford Biobank to participate in this study

# Do I have to take part?

- No, taking part in this study is entirely voluntary
- Any participant can withdraw from the study at any time, without giving a reason, even after giving consent
- Electing not to take part in this study, or changing your mind and withdrawing from this study will have no effect on your participation in the Oxford Biobank cohort

If you are interested in taking part, you will have the opportunity to ask questions and discuss the study in more detail. If you agree to take part, a researcher will ask you for your consent via an online form during a call with one of the members of our research team. The study will not proceed until consent has been given.

# What will happen to me if I decide to take part?

There are four primary aspects to this study which will take place over the course of a three day, four night measurement window:

1. You will undergo at-home sleep assessment to assess your sleep quality, duration, and time spent in different stages of sleep,
2. You will participate in physical activity monitoring by wearing activity trackers placed on your wrists, the front of your right thigh, your hip/waist and on your ankle,
3. Your heart function will be monitored using a small wearable electrocardiogram (ECG) adhered to your chest, and
4. Your activities of daily living will be logged using a forward-facing, first-person perspective video camera, worn clipped onto your clothing, capturing video footage during your waking hours.

Upon expressing interest in this study to the Oxford Biobank research team, your contact information will be provided to the OxWEARS research team. A member of the OxWEARS team will contact you to arrange a telephone call or online video call to answer any further questions you have, to ensure your eligibility for the study, and to receive your consent to enrol in this study. At no point will you be requested to make an in-person visit to the research offices; however, participation will require a visit by the researchers to your home to set up the devices for your in-home sleep assessment.

**In-Home Sleep Assessment**

At the beginning of your data collection period, 1-2 researchers will meet you in your home during the evening hours to set up your study. You will be asked to be wearing comfortable night clothes prior to the researcher’s arrival. You may also be asked to shave a portion of your chest or thigh prior to the visit to ensure good contact of the sensor’s adhesive with your skin.

When the researchers arrive at your home, they will prepare you for your in-home sleep study. Polysomnography (PSG - sleep study) will be recorded during the screening night to assess your sleep. Polysomnography (PSG) will include an EEG for the duration of the night. EEG stands for Electroencephalogram and it is the commonest form of measuring electrical activity of the brain. These are small electrodes placed on your scalp and to establish electrical contact between the scalp and the sensors, gel containing salts that conduct electricity would be placed under each metal contact. It is often necessary to prepare the area of the scalp under the sensor by cleaning it with rubbing alcohol and massaging an abrasive substance using a cotton swab, or by scratching the surface of the scalp with a blunt wooden stick. We would ask you to let the researcher know if at any time the procedure becomes uncomfortable. In such a case, we would stop the study procedure, without this having any negative consequences for you. The gel used to make the electrical contact is water based and washes away easily. In the morning following this first night, a researcher will again visit your home to collect this EEG equipment and assess your data.


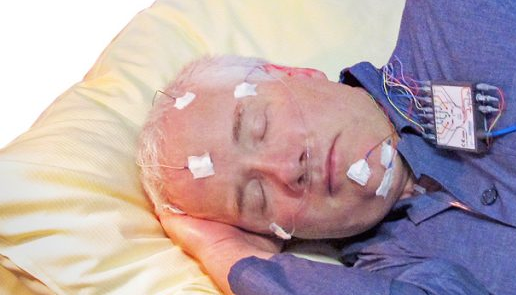


**Figure 1:** At-home polysomnography system with cranial and facial leads for use during first night of measurement (image adapted from <https://somnomedics.de>)

Your sleep study will only assess one night of sleep, however we are interested in your sleep habits throughout the measurement period. Every morning of the study we will ask you to fill out a short sleep diary that will ask you question about how much time you spent in bed and how well you slept.

**Sleep mat**

During the first visit to your home for the in-home sleep assessment, the researchers will additionally set up a sleep mat to measure your sleep. This mat is placed under the mattress of your bed, approximately in-line with where your heart would be whilst you are in bed. The sleep mat needs to be plugged into a power source throughout the study, though it uses a very small amount of electricity during this time. This mat relies on pressure sensors to detect when you are in bed, how much you move during sleep, and your heart rate. No personal information about you is inputted into the device, and any data remains on the device until the researcher collects it at the end of the study with your other equipment. You should not typically feel the mat underneath your mattress, however if the device causes you discomfort or disturbs your sleep, we would ask you to simply remove the device from under your mattress; this will not affect your participation in the remainder of the study.


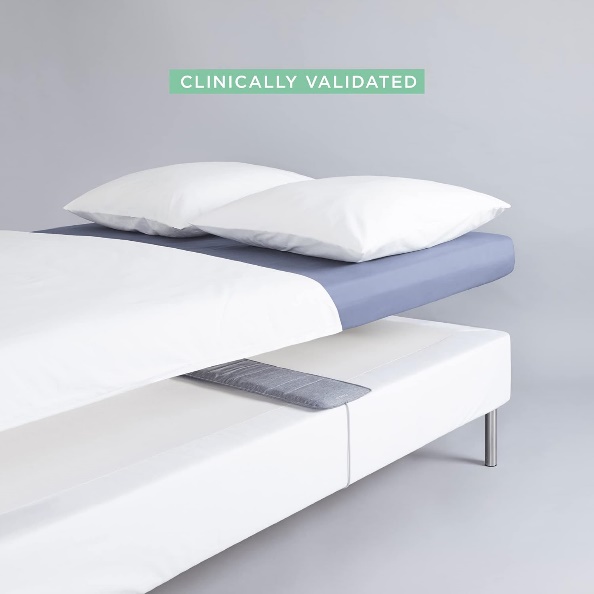


**Figure 2:** Sleep mat placed under mattress to detect sleep throughout the 4 nights of the study

**Activity Trackers**

When the researchers visit your home the first night, they will additionally set you up with five activity trackers to monitor your physical activity across different body locations; dominant wrist, non-dominant wrist and dominant-side hip, thigh and ankle (see Figure 3). These trackers are waterproof and can be worn 24 hours per day over the entire course of the study. These trackers record the amount of movement you are undertaking throughout the day. They are not set up to record GPS location, cannot track your whereabouts, and do not transmit any data in real time. If you have a smartphone, we will also ask you to record the number of steps your smartphone measures each day.

**
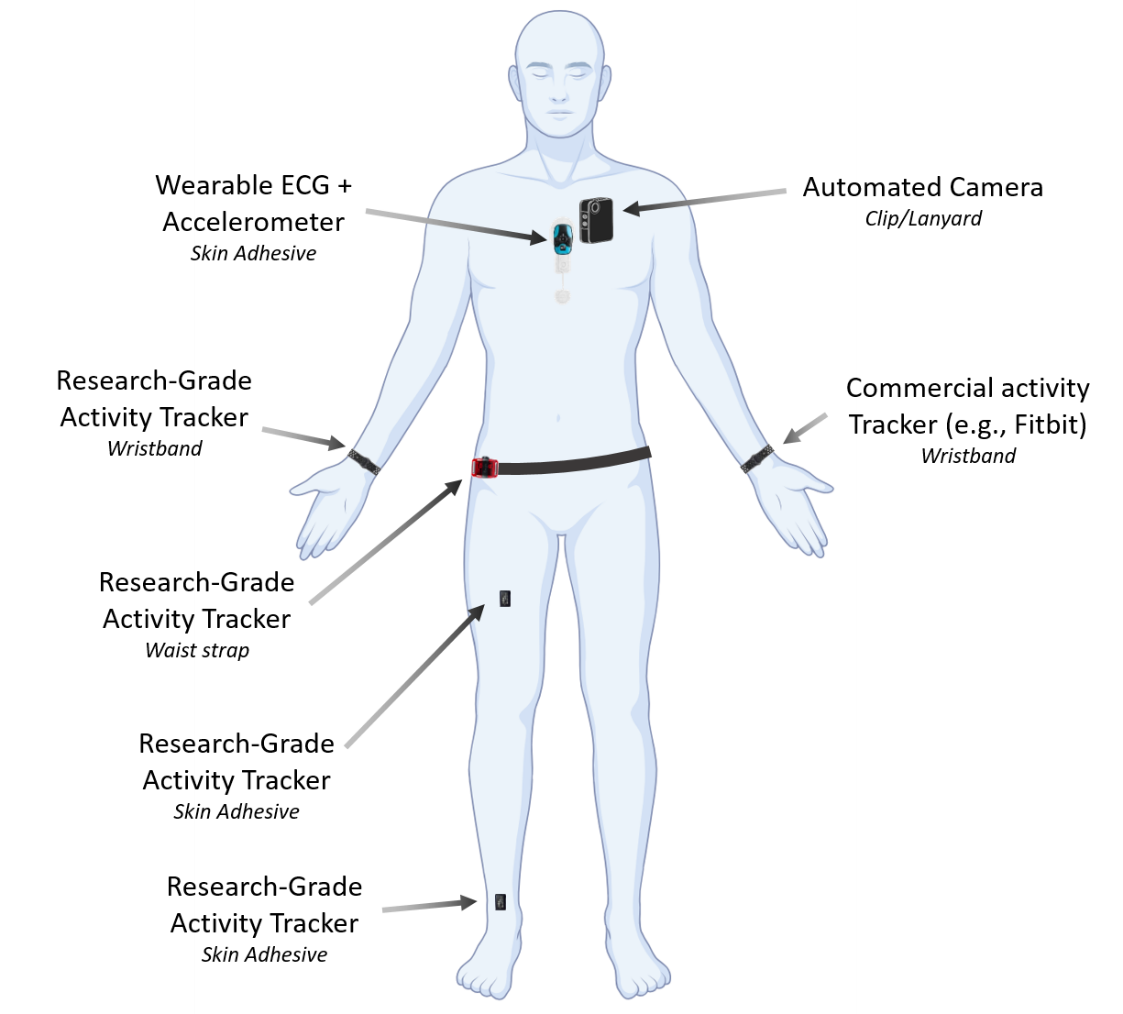
**

**Figure 3:** Distribution of wearables sensors for ambulatory monitoring, including wrist and thigh accelerometers, a chest-mounted ECG/accelerometer patch, and a video camera. The hip, thigh and ankle monitors are placed on your dominant side.

**Heart Monitoring**

You will also wear a chest-mounted heart monitor. This device will be worn 24 hours per day and is mounted on your chest using a medical-grade adhesive. This monitor is fully waterproof and will not need to be removed during bathing or swimming (unless at a depth of greater than 3 feet). If hair is present, you will need to shave the centre area of your chest prior to monitor placement.


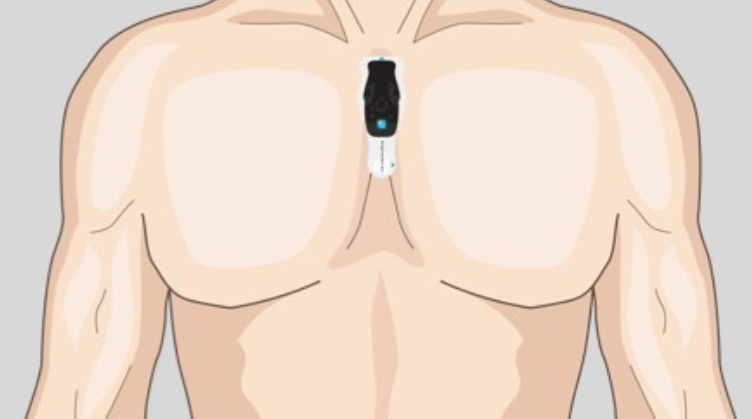


**Figure 4**: A wearables electrocardiogram similar to the one shown, will be placed on the sternum (breastbone) cardiac monitoring, sleep, and activity monitoring.

**Video Camera**

During your waking hours each day, you will be asked to wear a first-person perspective video camera, similar to the one in Figure 4. This camera records video and is designed to help the researchers understand the link between your activity tracker data and what activity you are participating in. The cameras do not record any audio. You will be able to remove or cover the camera at any time when you would like during any situations you feel uncomfortable, would like more privacy, or may not be appropriate for a camera (i.e. in a gym changing room). Data is logged and encrypted on the device, so only the research team will be able to recover the video after you have returned the camera at the end of the study.


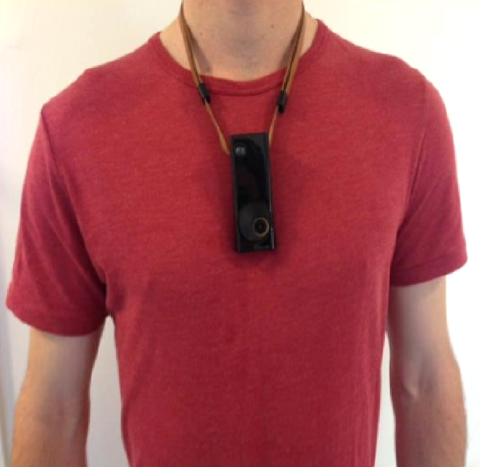


**Figure 5**: A chest-mounted video camera, similar to the one pictured above, will be worn by participants to log physical activity behaviours for all waking hours each day of study participation.

# What should I consider?

The study team will assess your eligibility for this study. You should not volunteer for the study if you have a skin sensitivity to metal or adhesives. You should carefully consider whether this study is appropriate for you if you are employed in a healthcare or childhood educational setting where wearing a camera may not be appropriate.

*Please note that assurances on confidentiality will be strictly adhered to unless evidence of wrongdoing or potential harm is uncovered. In such cases the University of Oxford may be obliged to contact relevant statutory bodies/agencies.*

# Are there any possible disadvantages or risks from taking part?

The sensors and electrodes for the sleep study are commonly used in sleep research and are non-invasive and only temporary attached to the skin of the subject. EEG is a procedure for measuring brain waves. It is harmless and painless and carries no significant risk to participants. EEG recording has been used safely for many years and we are aware of no cases of adverse events. EEG equipment comes from certified suppliers of medical equipment, who are obliged by law to adhere to published guidelines on electrical and mechanical safety (IEC-601). If you feel any discomfort, then please let the researcher know and they will stop the procedure. Slight irritation and abrasion of your skin can occur due to the cleaning and preparation required.

# What are the possible benefits of taking part?

You will receive a summary report about your physical activity and sleep after you have returned all of the sensors and your data has been analysed. By taking part in this study you are helping researchers to better understand how physical activity and health markers can be captured using wearables sensors. This information will be applied in further studies to associate activity and sleep behaviours with risk of diseases like cardiovascular disease and dementia.

# Will my General Practitioner/family doctor (GP) be informed of my participation?

Your GP will not be notified of your participation in this study, as your data will not be evaluated by a trained clinician. No clinical diagnoses or assessments of your health will be conducted in this study.

# Will my taking part in the study be kept confidential?

# Your participation in this study is strictly confidential and your identity will remain separate from the recorded physical activity data. At the onset of the study, you will be given a unique ID to which your research data will be logged with. The key tying your name to your activity data will be kept in an encrypted document a University of Oxford secure server. Once your data is collected and anonymized, it will not be possible for you to withdraw your data from the study. At the end of the study, deidentified sensor data will be made available to be shared with other researchers across the world for research use. Camera images will contain identifiable information and cannot be anonymized, thus will only ever be seen by members of the research team and never shared externally.

Responsible members of the University of Oxford may be given access to data for monitoring and/or audit of the study to ensure that the research is complying with applicable regulations.

# Will I be reimbursed for taking part?

You will not be reimbursed for participation in this study, but we will provide you with feedback on your sleep and activity for the measurement period.

# What will happen to my data?

Data protection regulation requires that we state the legal basis for processing information about you. In the case of research, this is ‘a task in the public interest.’ The University of Oxford is the data controller and is responsible for looking after your information and using it properly. The information that you provide through wearable sensors, video cameras, and keeping a sleep diary will be considered research data and will be kept separate from the personal data that we collect during study registration. Video files from the video camera will be analysed manually and through automated processes to classify activity behaviour. Research data will be deidentified and kept on a secure, password-protected University of Oxford storage server. Video footage will not be able to be deidentified, but will only be accessible by the OxWears research team. Physical copies of your personal contact data will be kept in a locked location on site at the NDPH Big Data Institute. The link codes, connecting the personal and research data, will be kept on paper with the personal data in a locked location at the NDPH Big Data Institute. Professor Aiden Doherty will be the custodian of all personal and research data, including consent forms and participant demographics, with personal data only available to members of the research team. Each member of the research team is obligated to maintain your confidentiality as a research study participant. Further information about your rights with respect to your personal data is available at <https://compliance.web.ox.ac.uk/individual-rights>. At any time, you can update your personal information or find out more about how we use your information by contacting [aiden.doherty@ndph.ox.ac.uk](mailto:aiden.doherty@ndph.ox.ac.uk).

# [What will happen if I don't want to carry on with the study?](http://hra-decisiontools.org.uk/consent/content-sheet-support.html#two)

Participation is voluntary and you may change their minds at any point of the study. Withdrawal will not affect your status as a participant in the Oxford Biobank study cohort. If you withdraw from the study, we will destroy all your identifiable samples, but will use the data collected up to your withdrawal.

# What happens at the end of the study?

# The results of this study will be available after it finishes and will usually be published in the form of a scientific journal article and/or scientific conference presentation. Deidentified sensor data will be made available for use by other global researchers. All research data will remain anonymous and participants will not be identified. video footage from wearable cameras will never be shared outside of the research group. Following the study, you will be provided with a summary of your physical activity and sleep patterns as derived from the sensor data.

# Your sensor data, including sleep study and heart monitor data will not be assessed by a clinician and will therefore not be screened for, or include, any feedback related to any potential medical concerns.

# What if there is a problem?

If you wish to complain about any aspect of the way in which you have been approached or treated, or how your information is handled during the course of this study, you should contact Professor Aiden Doherty (01865 617794, [aiden.doherty@ndph.ox.ac.uk](mailto:aiden.doherty@ndph.ox.ac.uk)) and we will do our best to answer your query. We will acknowledge your concern within 10 working days and give you an indication of how it will be dealt with. If you remain unhappy or wish to make a formal complaint, please contact the University of Oxford Research Governance, Ethics & Assurance (RGEA) team at [rgea.complaints@admin.ox.ac.uk](mailto:rgea.complaints@admin.ox.ac.uk) or on 01865 616480.

# How have patients and the public been involved in this study?

Eight members of the public were involved developing the data collection protocol, taking into consideration the frequency of study visits, number of sensors, and planned communication between participants and the research team.

# Who is organising and funding the study? This study is being organised by Aiden Doherty, Professor of Biomedical Informatics in the Nuffield Department of Population Health at the University of Oxford. The study is funded by the Wellcome Trust.

# Who has reviewed the study?

This study has been reviewed by, and received ethics clearance through, the University of Oxford Central University Research Ethics Committee (Reference: R74559/RE001.

# Further information and contact details:

If you have additional questions or concerns, please contact Professor Aiden Doherty via email at aiden.doherty@ndph.ox.ac.uk.
