# Supplementary material for: Generation of a Free-Living Ground-Truth Validation Dataset for Wearable Measures of Physical Activity, Sedentary Behavior, Sleep, and Heart Rate in Adults (OxWEARS): Protocol for a Cross-Sectional Study
Source: JMIR Res Protoc. 2025 Dec 29;14:e78779. doi: 10.2196/78779 (PMC12747664; doi:10.2196/78779)
Supplement: Multimedia Appendix 2 [file resprot-v14-e78779-s002.docx]

**Equipment and Privacy Agreement**

We are interested in measuring your physical activity, heart function, and sleep using wearable sensors. You will be wearing a combination of devices, including highly sensitive scientific technology. Further you will be wearing a front-facing video camera designed specifically for this study. Before participating in this study, we require your agreement with the following conditions:

**Camera**

- Your wearable camera will record video from your perspective. In other words, everything that you see in front of you during the day could potentially be recorded.
- Please make every effort not to invade others’ privacy. This includes removing/covering the camera when entering a gym changing room, toilet, school, hospital, or other sensitive location. If possible, please check with friends, family, and work colleagues before wearing the camera in their presence. It is better to temporarily remove or obscure the camera than to risk making a bystander uncomfortable or angry.
- Please be immediately upfront with others that you are wearing a camera, and that you are happy to remove it if they desire, saying: **“I am volunteering for a research study about my everyday activities. This is a wearable camera. It records video but not sound or conversations. I would be happy to remove it if you would like me to.”**
- Please be aware that any intentional video capture of illicit or inappropriate behaviour will require reporting to the University of Oxford ethics team, and potentially to appropriate authorities.

**Sleep Study Equipment**

- Sleep study equipment is highly specialized medical equipment with a high replacement cost. **Sleep study equipment is not waterproof.** Once your sensors have been placed, please do not shower or bathe until you have removed all sensors from your head and face the next morning.

**All Equipment**

*All equipment is the property of the University of Oxford and must be returned in good faith following the completion of the study.*

Please confirm that you have read and understood the equipment and privacy agreement instructions:

Name: _______________________________ Signature: ____________________________________

If you have any questions about the equipment in this study please contact:

Professor Aiden Doherty

[aiden.doherty@ndph.ox.ac.uk](mailto:aiden.doherty@ndph.ox.ac.uk)

01865 617794
