# Supplementary material for: Generation of a Free-Living Ground-Truth Validation Dataset for Wearable Measures of Physical Activity, Sedentary Behavior, Sleep, and Heart Rate in Adults (OxWEARS): Protocol for a Cross-Sectional Study
Source: JMIR Res Protoc. 2025 Dec 29;14:e78779. doi: 10.2196/78779 (PMC12747664; doi:10.2196/78779)
Supplement: Multimedia Appendix 3 [file resprot-v14-e78779-s003.docx]

|  | **Sample** | **Night 1 (Sleep Study)** | **Night 2** | **Night 3** | **Night 4** |
| --- | --- | --- | --- | --- | --- |
| Today’s date | 4/5/11 |  |  |  |  |
| 1. What time did you get into bed? | 10:15 pm |  |  |  |  |
| 2. What time did you try to go to sleep? | 11:30 pm |  |  |  |  |
| 3. How long did it take you to fall asleep? | 55 min. |  |  |  |  |
| 4. How many times did you wake up, not counting your final awakening? | 3 times |  |  |  |  |
| 5. In total, how long did these awakenings last? | 1 hour 10 min. |  |  |  |  |
| 6. What time was your final awakening? | 6:35 am |  |  |  |  |
| 7. What time did you get out of bed for the day? | 7:20 am |  |  |  |  |
| 8. How would you rate the quality of your sleep? | - Very poor - Poor - Fair - Good - Very good | - Very poor - Poor - Fair - Good - Very good | - Very poor - Poor - Fair - Good - Very good | - Very poor - Poor - Fair - Good - Very good | - Very poor - Poor - Fair - Good - Very good |
| 9. Did you take any naps during the day? (If yes, please specify start/end time*)* | 16:00 to 16:45 |  |  |  |  |
| 10. Comments  (if applicable) | I have a cold |  |  |  |  |
